# “Educational Material on HIV”: validity of health educational technology for people living with HIV

**DOI:** 10.1590/0034-7167-2022-0549

**Published:** 2023-08-07

**Authors:** Ana Carolina Souza de Lima, Blenda Gonçalves Cabral, Jaqueline Dario Capobiango, Marcos Hirata Soares, Flávia Meneguetti Pieri, Gilselena Kerbauy

**Affiliations:** I Universidade Estadual de Londrina. Londrina, Paraná, Brazil

**Keywords:** Educational Technology, Validation Study, Health Education, Acquired Immunodeficiency Syndrome, HIV., Tecnología Educacional, Estudio de Validación, Educación en Salud, Síndrome de Inmunodeficiencia Adquirida, VIH., Tecnologia Educacional, Estudo de Validação, Educação em Saúde, Síndrome da Imunodeficiência Adquirida, HIV.

## Abstract

**Objectives:**

to validate the educational technology “Educational Material on HIV” (INPI - BR 10 2020 003765 0).

**Methods:**

a methodological study with 39 expert judges in HIV/AIDS, using a 5-point Likert scale for assessment. Data were tabulated, processed and analyzed through descriptive analysis. Cronbach’s alpha and McDonald’s omega tests were performed to analyze internal consistency, and the Intraclass Correlation Coefficient, for reliability. Agreement was established by a Level Content Validity Index greater than 0.90.

**Results:**

the assessment instrument showed high internal consistency (Cronbach’s alpha of 0.89; McDonald’s omega of 0.91) with reliable values. Based on the Intraclass Correlation Coefficient, judges’ answers showed acceptable reliability, mean score 0.89 (p<0.001). Agreement among judges was greater than 0.90 in the three assessed dimensions (objectives, presentation and relevance).

**Conclusions:**

the technology was considered a qualified and adequate tool by the judges regarding its objectives, presentation and relevance.

## RESUMO

**Objetivos:**

validar a tecnologia educacional “Material Educativo sobre HIV” (INPI - BR 10 2020 003765 0).

**Métodos:**

estudo metodológico com 39 juízes *experts* em HIV/AIDS, utilizando escala Likert de 5 pontos para avaliação. Os dados foram tabulados, processados e analisados mediante análise descritiva. Realizaram-se os Testes alfa de Cronbach e ômega de McDonald para análise da consistência interna, e *Intraclass Correlation Coefficient*, para confiabilidade. A concordância foi estabelecida pelo *Level Content Validity Index* maior que 0,90.

**Resultados:**

o instrumento de avaliação apresentou alta consistência interna (alfa de Cronbach de 0,89; ômega de McDonald de 0,91) com valores confiáveis. A partir do *Intraclass Correlation Coefficient*, as respostas dos juízes apresentaram confiabilidade aceitável, escore médio 0,89 (p<0,001). A concordância entre os juízes foi superior a 0,90 nas três dimensões avaliadas (objetivos, apresentação e relevância).

**Conclusões:**

a tecnologia foi considerada uma ferramenta qualificada e adequada pelos juízes quanto aos seus objetivos, apresentação e relevância.

## RESUMEN

**Objetivos:**

validar la tecnología educativa “Material Educativo sobre VIH” (INPI - BR 10 2020 003765 0).

**Métodos:**

estudio metodológico con 39 jueces expertos en VIH/SIDA, utilizando una escala Likert de 5 puntos para evaluación. Los datos fueron tabulados, procesados y analizados mediante análisis descriptivo. Se realizaron las pruebas alfa de Cronbach y omega de McDonald para analizar la consistencia interna y el *Intraclass Correlation Coefficient* para la confiabilidad. La concordancia fue establecida por un *Level Content Validity Index* superior a 0,90.

**Resultados:**

el instrumento de evaluación mostró alta consistencia interna (alfa de Cronbach de 0,89; omega de McDonald de 0,91) con valores confiables. Con base en el *Intraclass Correlation Coefficient*, las respuestas de los jueces mostraron una confiabilidad aceptable, puntaje promedio de 0,89 (p<0,001). La concordancia entre jueces fue superior a 0,90 en las tres dimensiones evaluadas (objetivos, presentación y relevancia).

**Conclusiones:**

la tecnología fue considerada una herramienta calificada y adecuada por los jueces en cuanto a sus objetivos, presentación y pertinencia.

## INTRODUCTION

The global health policy aimed at people living with human immunodeficiency virus (PVHIV) proposes the continuous care cascade, a health care strategy composed of sequential steps in care that begin with the timely diagnosis of people infected with human immunodeficiency virus (HIV), followed by linking this individuals to the health service, their retention in clinical and laboratory follow-up, prompt initiation of treatment and promotion of antiretroviral therapy (ART) compliance to achieve viral suppression and quality of life^(1)^.

Promoting self-care through health education from patients’ first link to the health service and sustaining this process throughout treatment, supporting strategies of individualized reception, active listening and guidance aimed at health promotion, is essential^(2)^. In this regard, innovative educational technologies that illustrate and demonstrate the content addressed to the target audience are considered the most appropriate to facilitate and contribute to their educational process^(3-4)^.

Despite the existence of several educational technologies validated for this audience^(4-9)^, most are booklets that gather information about acquired immunodeficiency syndrome (AIDS) without the intention of using them together with PLHIV during care in health services. Furthermore, in the Unified Health System (SUS - *Sistema Único de Saúde*), there is no technological tool for standardized and recommended educational actions to support health professionals during teaching-learning strategies on the subject.

In order to improve care and support health education actions for PLHIV, a broad search of educational technologies was carried out in national and international patent databases, but no effective health education methodology for this audience was found. Therefore, based on the professional experience of a nurse and a professor in specialized care service and in the hospitalization sector for HIV/AIDS, an educational technology in health was developed over the course of 2018 and 2019.

The technology development was based on national and international theoretical frameworks on HIV/AIDS^(10-12)^. Technology prototypes were tested for 12 months, which led to advances and adaptations to the material. In 2020, a patent for the invention called “Educational Material on HIV” (*Material Educativo sobre HIV*) (Patent Deposited/Brazilian National Institute of Industrial Property (INPI - *Instituto Nacional da Propriedade Industrial*) - BR 10 2020 003765 0) was requested by the *Universidade Estadual de Londrina* (UEL).

The educational technology in health was made of plastic material and composed of pieces that illustrate the bloodstream, lymphocytes (LT-CD4+), HIV virus, HIV strains resistant to treatment, pills that represent ART and the action of this therapy. The technology is an illustrative, didactic, dynamic and interactive method that uses the movement of pieces together between a health professional and PLHIV to explain the natural cycle of virus infection, the development of AIDS, the action of ART, the achievement of viral suppression through adequate and continuous treatment compliance as well as the development of viral resistance due to treatment non-compliance. A demonstration of use of this material is available on video^(13)^.

Although the technology is innovative and necessary for promoting health education, materials for application in care need assessment and validity by expert professionals^(14)^.

Considering the health policy for PLHIV and the proposition of an educational technology, the following question was asked: is the “Educational Material on HIV” (INPI - BR 10 2020 003765 0) valid and applicable for carrying out health education in consultations to PLHIV according to expert professionals?

## OBJETIVOS

To validate the educational technology “Educational Material on HIV” (INPI - BR 10 2020 003765 0) content by expert judges in the field of infectiology with experience in the HIV/AIDS theme.

## METHODS

### Ethical aspects

The study complied with the precepts of Resolution 466/2012 of the Brazilian National Health Council, and was approved by the Research Ethics Committee at UEL. Consent was obtained from all individuals involved in the study through the Informed Consent Form (ICF) on the Google Forms^®^ platform.

### Study design, period, and place

This is a methodological study to validate the “Educational Material on HIV” content, which followed the Guidelines for Reporting Reliability and Agreement Studies (GRRAS)^(15)^ recommendations. The study was carried out in two stages: 1) Structuring and elaboration of audiovisual content to demonstrate the technology and the instrument used by judges to validate the technology attributes; 2) Validity by equivalence of educational technology by judges with expertise in infectiology with an emphasis on the HIV/AIDS theme. Data collection took place from January to March 2021, online, with the aim of covering graduated health professionals throughout the national territory.

### Population and sample: inclusion and exclusion criteria

Atudy population recruitment took place using a non-probabilistic snowball sampling system, which was initiated and propagated via WhatsApp^®^, Facebook^®^ and Instagram^®^. The study population consisted of 84 professionals, and to obtain the sample of judges, Fehring^(16)^ criteria adapted for the area of infectiology and HIV/AIDS were used.

These criteria cover professional training data, including *lato* and *stricto sensu* graduate studies, scientific production and professional experience. The maximum score of the criteria reaches 14 points. However, according to recommendations^(16)^, professionals with a score equal to or greater than 5 were included in the sample, with a sample consisting of 39 judges.

### Study protocol

The instrument used for assessment was adapted^(17)^ regarding judges’ characterization according to Fehring’s criteria^(16)^, inclusion of educational technology characteristics in all instrument items, addition of a point of agreement on a Likert scale^(18)^ and exclusion of an item that refers to the identification of material pieces, after analysis of the instrument’s internal consistency.

Therefore, the instrument was composed of two corresponding sections: section 1 - judge characterization; section 2 - 22 assessment items subdivided into three domains (objectives, presentation and relevance) scored by a Likert scale with five points of agreement: totally agree, partially agree, indifferent, partially disagree and totally disagree. At the end of each item, an optional space was provided for judges’ notes to assist in adapting the technology. These textual components were grouped by domain and presented in full in Table 1 in the results section.

**Table 1:** Judges’ assessment regarding domains and items of educational technology for people living with human immunodeficiency virus (N=22), Londrina, Paraná, Brazil, 2021

Educational material domains and items	Levels of agreement					
	TA	PA	I	PD	TD	I-CVI
1 Objectives						
1.1 Contemplates the proposed theme	38	1				1.00
1.2 The information conveyed by educational technology is adequate	37	2				1.00
1.3 Provides reflection on the topic	36	3				1.00
1.4 Encourages treatment compliance	35	4				1.00
1.5 Appropriate for PLHIV’s teaching-learning	38	1				1.00
2 Presentation						
2.1 Clarifies about HIV infection and development of AIDS	36	3				1.00
2.2 Illustrates antiretrovirals’ activity	36	2		1		0.97
2.3 Addresses HIV resistance	36	2		1		0.97
2.4 Explain the condition of undetectable viral load	38	1				1.00
2.5 Has a logical sequence in infection and treatment presentation	37	2				1.00
2.6 Information is presented in an objective manner	38	0		1		0.97
2.7 Educational technology allows interaction between professional and target audience	38	1				1.00
2.8 Enables using adequate and accessible language for health education for PLHI	34	5				1.00
2.9 The material is appropriate for the different sociocultural levels of the target audience	32	7				1.00
2.10 The pieces that make up the technology are relevant to the content demonstrated	37	2				1.00
2.11 The pieces are easy to see	38	0		1		0.97
3 Relevance						
3.1 Provides knowledge on HIV	38	1				1.00
3.2 Arouses interest in the subject in the target audience	35	4				1.00
3.3 Motivating and encouraging content for the target audience	36	3				1.00
3.4 Applicable in health professionals’ practice to care for PLHIV	37	2				1.00
3.5 Contributes to the target audience’s knowledge	36	2	1			0.97
3.6 Has the potential to be used in healthcare institutions	37	2				1.00
S-CVI/Ave						0.99

*HIV - human immunodeficiency virus; AIDS - acquired immunodeficiency syndrome; PLHIV - people living with HIV; TA - totally agree; PA - partially agree; I - indifferent; PD - partially disagree; TD - totally disagree; I-CVI - Level Content Validity Index (item level); S-CVI/Ave - Scale-Level Content Validity Index/Average Calculation Method (general instrument level).*

The questionnaire was formulated in Google Forms^®^, containing a letter of material presentation with guidelines on validity, a demonstrative video of material application^(13)^ to be assessed and the ICF, with a return period of 60 days. The educational technology was demonstrated through audiovisual content, which exposes the actual simulation of material use through the performance of actors playing patients and health professionals. Audiovisual production was carried out with the support of Photoshop CC 2019^®^ and Wondershare Filmora 9^®^ programs.

### Analysis of results, and statistics

The judges were characterized through descriptive analysis (frequency distribution and measures of central tendency). The instrument data were analyzed using Cronbach’s alpha (α) and McDonald’s omega (Ω). Validity data were obtained from judges’ answers on a Likert scale and were analyzed for inter-judge reliability by the Intraclass Correlation Coefficient (ICC), using the bilateral combined randomization model.

To measure the agreement among judges on the educational technology attributes, the Level Content Validity (CVI - Content Validity Index) was used, calculated in two procedures: Level Content Validity Index (I-CVI), which assessed the level of agreement among expert judges regarding each item, through the number of judges who rated the item as “totally agree” and “partially agree”, divided by the total number of judges; and Scale-Level Content Validity Index/Average Calculation Method (S-CVI/Ave), which calculated the mean I-CVI of the 22 assessed items as well as the mean I-CVI of items in each domain. Items with a level greater than 0.90 were considered valid^(19)^.

Data come from a master’s thesis in nursing and were tabulated in Microsoft Excel^®^, analyzed using IBM SPSS^®^ Statistics, version 26.0 (International Business Machines Corporation, Armonk, New York, USA), and Jamovi^®^, version 1.8. 4.0, adopting a significance level of 5% (p-value <0.05).

## RESULTS

The sample consisted of 39 judges with expertise in infectiology with an emphasis on HIV/AIDS, who had a Fehring criteria average score of 7.71 points (SD 2.70). The judges came from all regions of Brazil, with a predominance of judges from the South (58.97%), the Northeast (20.51%) and the Southeast (12.82%). The mean age was 47.74 years (SD 10.73), ranging from 26 to 67 years, with a predominance of females (87.18%).

The most frequent professional categories were nursing (58.97%), medicine (10.26%) and social work (10.26%), with predominance of work in teaching (48.72%), health care (46.15 %) and health service management (28.21%), in addition to more than 10 years of experience in infectiology and HIV/AIDS (51.28%). There was a predominance of judges with a *stricto sensu* graduate degree, with 28.21% holding a master’s degree and 38.46% holding a doctoral degree, with scientific publications in infectiology and HIV/AIDS (64.10%).

The technology validity instrument showed high internal consistency among its items (Cronbach’s α 0.89; McDonald’s Ω 0.91), being considered reliable for application. All instrument items did not present any damage to internal consistency, maintaining the 22 items in the scale.

For the educational technology validity, an assessment round was carried out, where ICC analysis of judges’ answers presented a score with an average value of 0.89 (p<0.001), suggesting acceptable reliability. With regard to agreement among judges, options “partially agree” and “totally agree” were marked by 100% of judges in 18 items and 97.4% in 5 items. Among all items, none had an I-CVI lower than 0.90, with 99.4% having an I-CVI of 1.00 (Table 2). Therefore, it was not necessary to carry out a second assessment round of educational technology.

Judges’ levels of agreement in relation to the objective, presentation and relevance domains varied with a mean of 0.99 (SD 0.011), minimum level of 0.97 and maximum of 1.00 (Table 2). Among the 39 judges, the majority (94.8%) presented an I-CVI of 1.00 in relation to assessed items, and only one judge (2.56%) showed agreement below 0.90 in relation to all instrument items (0.87). However, the items’ overall mean reached the expected level of agreement (≥0.90) with an S-CVI/Ave of 0.99.

As for the comments made in an essay, 48.72% of judges wrote at least one opinion about educational material. Experts point out in their speeches the material relevance, indicating that the technology is relevant to the health education process during PLHIV care (Chart 1).

**Chart 1:** Transcription of judges’ comments according to the domain of validity contents of educational technology for people living with human immunodeficiency virus, Londrina, Paraná, Brazil, 2021

Domain	Judges’ comments regarding educational technology
Objectives	*- Simple application material* [...] *facilitates patients’ understanding and makes them more involved in treatment. Patients more easily understand the importance of compliance* (J3); *- Compliance is a crucial issue in PLHIV treatment and this educational material will certainly contribute to this educational work (J5);* *- Easy understanding* (J6); *- Very creative and adequate to understanding. As it explains the form of treatment and the possibility of appearance of opportunistic diseases, as well as resistant forms, it favors better compliance to treatment by understanding the infectious process and its consequences when used irregularly. The presentation individually, as proposed by the technology, will favor interaction with individuals and resolve doubts that may arise during exhibition* (J7); *- Excellent tool for educational work, very simple, objective and didactic. We serve many people with low education and this can be another illustrative resource to contribute to the dialogue with users. Information structured in a didactic way, the importance of compliance to treatment is very clear. Encourages compliance as it shows that abandoning treatment can encourage the emergence of resistant viruses, requiring the introduction of other medications that can cause side effects to treatment* (J8); *- Very simple, objective and didactic* (J9); *- Demonstrates in a didactic way the relationship between the virus and TCD4 lymphocytes, demonstrating in an enlightening way the importance of antiretroviral treatment compliance. Remembering that, from the pedagogical point of view, when a learning object is completely new to students, concretizing is more effective than explanations from abstractions. Understanding the consequences of non-compliance encourages treatment* [...] *makes participants realize relevant questions about the pathology* (J10); *- Didactic material and easy to understand. Adequate information* (J11).
	*- Easy comprehension/understanding,* [...] *it makes you reflect and not give up promptly* (J14); *- The material materializes the theory,* [...] *it helps to materialize the effects of drugs and knowledge.* [...] *in a simple way, it makes it easier to understand the theory. From the complex to knowledge and to change behavior and thus make individuals more capable of taking conscious actions* (J22); *- The material is really cool and I think it will contribute a lot to PLHIV self-care* (J27); *- Clear information, easy to understand* (J31); *- Encourages compliance using playful education.* [...] *the explanation is clear and reaches the different cultural and intellectual levels of patients. It should be instituted in all HIV/AIDS outpatient clinics due to its simplicity of application and cost (J34);* *- Well-illustrated and objective material,* [...] *very good, congratulations on the initiative! It provides reflection on the subject as it shows patients the consequences of non-compliance to treatment. Encourages treatment compliance*(J35); *- Very didactic and easy to understand* (J37); - [...] *super didactic material, wonderful* [...]*, provides reflection on the subject and answers questions* (J39).
Presentation	*- Patients will better understand how lack of compliance implies resistance. Very easy for patients to understand complex concepts using the material.* [...] *the material is excellent and practical* (J3); *- Very good illustrations* (J7); *- The elements allow clarification and clearly illustrate the theme* [...] *success will depend on the dynamics instructor and his ability to dialogue with patients* (J10); *- Technology images strengthen the imagination* (J14); *- It makes the theory domain accessible to be shared* (J22); *- It depends on the language professionals use. It is appropriate for all sociocultural levels* (J26); *- The material is simple, easy and colorful, the way we like to explain it to our patient* (J39).
Relevance	*- The material will be useful and its use can encourage a more person-centered listening* (J2); *- Material use depends on the volume of patients that the institution serves* (J3); *- Great instrument for approaching PLHIV* (J7); *- If the public is motivated and sensitized to receive this new knowledge, it will be fantastic* (J20); *- Rich and easy to understand material* (J39).

*PLHIV - people living with human immunodeficiency virus; J - judge.*

As shown in Chart 1, judges’ comments converged in the field of technology objectives, indicating that using the material meets the health education objectives aimed at PLHIV. As for physical presentation, material characteristics that enhance understanding by the target audience were highlighted. Regarding technology relevance, judges highlighted the material usefulness, conditioning the demand for services and users’ motivation.

**Figure 1:**
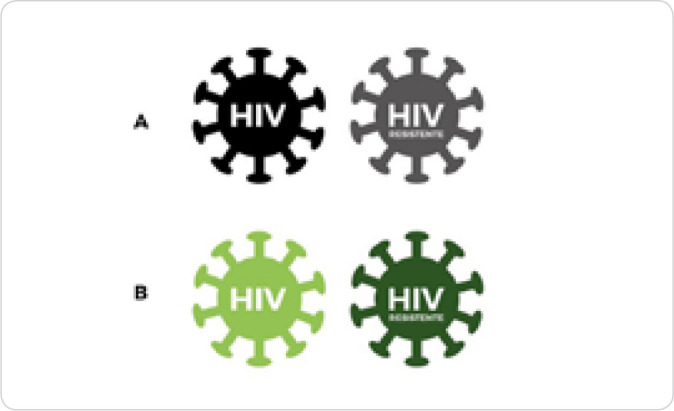
Illustration of sensitive and resistant virus pieces before and after content validity of “Educational Material on HIV”, Londrina, Paraná, Brazil, 2021

**Figure 2:**
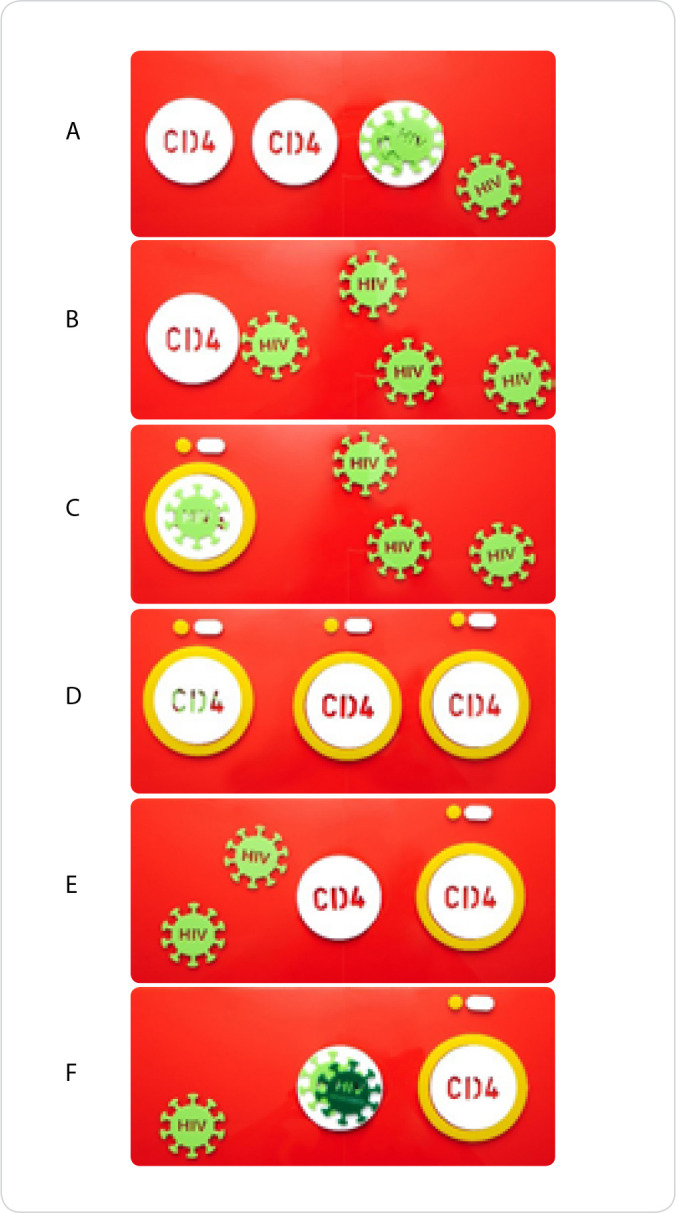
Presentation of the final version of “Educational Material on HIV” and representation of its use to demonstrate HIV pathophysiology and treatment, Londrina, Paraná, Brazil, 2021

There was only one suggestion directed towards the design of the resistant virus (Judge 7), “*How about removing some spikes from the resistant HIV? Or some other way to differentiate it more?*”. This suggestion was accepted in order to highlight the technology visualization, maintaining the shapes of the pieces, while the colors of sensitive and resistant viruses were changed (Figure 1). The other pieces of “Educational Material on HIV” remained identical to the version presented to the judges, since there was no suggestion for these components (Figure 2).

*A - HIV sensitive and HIV resistant pieces prior to content validity; B - HIV sensitive and HIV resistant pieces after content validity.*

*A - Beginning of HIV infection; B - Destruction of CD4+ T Lymphocytes (LT-CD4+) and development of AIDS; C - Antiretrovirals’ action (ARV); D - Achieving an undetectable viral load and immune reconstitution; E - Interruption of ARV use F - Selection of ARV-resistant virus strains.*

## DISCUSSION

The “Educational Material on HIV” is an innovative technology that was validated by judges with expertise in infectiology with an emphasis on HIV/AIDS from all regions of Brazil. The validity process included measuring educational material reliability, using the internal consistency analysis of the validity instrument, the equivalence and agreement of answers among judges.

The instrument used in validity showed high internal consistency, thus being homogeneous and reliable to measure the technology attributes^(14)^. Although alpha is widely used in psychometric studies, it was necessary to link the analysis of McDonald’s omega coefficient to bring precision and reliability to the results, since alpha was developed for one-dimensional instruments^(20)^, diverging from the instrument of this study.

Considering the reliability score of the study judges’ answers, it is inferred that the technology presented congruence and consistency in the assessed dimensions^(21)^. Judges’ agreement in relation to educational technology was higher than what is recommended in validity studies^(19)^ in all dimensions.

The comments pointed out by judges emphasize that the technology is simple, objective, didactic, creative and playful, as well as well illustrated, easy to apply and understand for PLHIV. The validity qualitative components reinforced the benefits of using technology to promote health education for PLHIV, corresponding to an essential step for validity^(3,6-7,9,22)^.

Educational actions on HIV, with the support of educational technologies, were identified as facilitators of improvement in individuals’ coexistence with the disease, patients’ bond with professionals and service^(5)^. PLHIV with lack of knowledge about the infection and treatment have low compliance to ART, in addition to self-stigma related to living with HIV^(23-24)^.

The comments denote that technology favors learning and materializes to patients what is being addressed during the educational process in an interactive, didactic and dialogic way. By supporting the pieces, this didactic resource facilitates subjects’ understanding addressed during health education^(25)^.

According to educational technology qualitative assessment, “Educational Material on HIV” is intended for use in health care settings, with the aim of promoting health education through the demonstration and interaction between professionals and users through piece handling, making the education process meaningful and effective. This pedagogical activity must be completed when all questions are resolved and, if necessary, the steps with handling the pieces can be repeated to clarify doubts.

The current global strategy of the Joint United Nations Program on HIV/AIDS, known as the “95-95-95” target, seeks to diagnose 95% of people infected with HIV, keep 95% of them on antiretroviral treatment and achieve viral suppression in 95 % of people on treatment seeking to end HIV infections and their complications by 2030^(1)^.

In line with global public policy to control HIV infection, validated educational technology can help achieve the aforementioned goals, considering its use in educational interventions at times when this patient is linked to the health service and throughout their clinical follow-up.

### Study limitations

Limitations of this study include the impossibility of sending the material in its physical form to the judges, due to the territorial expansion of the sample, and the lack of semantic validity of the instrument. However, considering the answers and comments expressed by judges about the educational technology, it is understood that the validity process, through the demonstrative video, was satisfactory.

### Contributions to nursing

This educational technology can assist in health promotion practices by nursing and other areas that permeate PLHIV care during teaching-learning strategies about HIV/AIDS in patients’ bond with the health service and during their follow-up, helping to clarify doubts inherent to the theme.

It is expected that the technology will be widely used in health services to support the aforementioned actions, given that in SUS there is no standardized technological tool available and recommended to support the actions of nursing and other health areas in PLHIV care.

## CONCLUSIONS

The innovative technology was validated by expert judges in three dimensions that encompass the material objectives, presentation and relevance. The study considers “Educational Material on HIV” an excellent strategy, with the potential to fill the existing gap of educational materials in health services, noting the lack of innovative demonstrative and interactive technologies for PLHIV. Future studies will be carried out for validity with the target audience as well as others aimed at the impact of using educational material on PLHIV’s lives.
